# Case of a Tumor, Apparently Cancerous, Disappearing Suddenly

**Published:** 1860-03

**Authors:** T. B. Cox

**Affiliations:** Kirklin, Ind.


### ARTICLE 3.

### CASE OF A TUMOR,

### APPARENTLY CANCEROUS, DISAPPEARING SUDDENLY.

BY T. B. COX, M. D., OF KIRKLIN, IND.

Mr. M. P——, æt. 40, presented himself at my office, to consult me respecting a tumor in the inguinal region, of three months standing.

*Present appearance.*—Has attained considerable size, extending nearly across the inside of the femur, and longitudinally some six inches; the upper margin being rather illy defined. Nothing unusual in external appearance; rather a bluish cast; no enlarged veins to be seen; soft and elastic to the touch.

*Previous History.*—On first making its appearance, was supposed to be an abscess, consequently was poulticed and lanced; instead, however, of pus, a little dark blood oozed from the opening.

*General Health*—Not good; skin rather a sallow hue; appetite moderate; bowels somewhat costive; has had several slight chills previous to this time, for which quinine was given with success. Fearing the malignancy of the disease, I postponed giving a definite answer as to the propriety of an operation, for which purpose I was consulted at the same time, guardedly hinting my fears to the patient; but being of rather a hopeful disposition, and having all his life enjoyed moderate health, he was slow to believe.

January 26th, met my friend Dr. C—— of Frankfort, at the residence of the patient. Appearance of the tumor not much altered; general health declining; slight fever, but a full flow of spirits, and a fixed determination to undergo an operation. At the suggestion of Dr. C., I introduced a probe, which passed easily through the opening without producing the least pain; but was followed by the loss of half a pint of blood, upon which the patient grew very faint, and became convinced that his health would not admit an operation.—Sulph. Quinia and Mur. Tinct. Ferri, were then prescribed in order, if possible, to improve his general health, though on our part with but little hope of any benefit.

February 1st, health not improved in the least. A fungus growth has made its appearance at the opening, resembling fungus hæmatodes, as described by Gibson. Gave a mild alterative and tonic treatment.

Feb. 16th. Tumor presents a ragged appearance; has bled but little; lymphatic glands considerably swollen, especially in the axillary region, and about the neck.

Now told him plainly that all hopes of benefitting him by an operation, were abandoned. Gave him full doses of Iodide Potass., four times per day.

February 27th. Patient rapidly declining; upper margin of the tumor better defined; prescribed Fowler's solution three times per day, in eight drop potions, alternated with sulph. quinæ.

March 2d. Considerable fever in the afternoon; tongue cracked; bowels tender; bloody stools; general symptoms, typhoid. Recipe—Turpentine emulsion at proper intervals, alternated with stimulants.

Suffice it to say that in a few days from this time, our patient died, with every vestige of the original tumor removed, and entire subsidence of all glandular swelling.

Was it fungus hæmatodes? It was surely some kind of soft cancer.

To my mind, the most interesting feature of the case, was the rapid recession of the tumor, and the consequent rapid decline of the patient. Was the deposit going on in some other organ? No symptoms of disease were detected in the lungs or heart. The spleen was, however, somewhat enlarged, but the patient lived in a malarious district, and had had ague. Was the tumor absorbed in the circulation, thereby acting as a poison?

